# Intermetallic Passivation Imparts Corrosion Resistance and Enables Intrinsic Electrocatalytic Activity Promotion

**DOI:** 10.1021/jacs.6c09853

**Published:** 2026-09-30

**Authors:** Majd M. Matalkeh, Jin Li, Andrew J. Wong, Alan Dang, Bryan D. Vogt, Michael J. Janik, Ezra L. Clark

**Affiliations:** Robert V. Waltemeyer Department of Chemical Engineering, 8082 The Pennsylvania State University, University Park, Pennsylvania 16802, United States

## Abstract

Electrocatalytic technologies are limited by reliance on precious metal catalysts with low activity. Intermetallics can alleviate these challenges but have limited efficacy due to corrosion susceptibility. Herein, we demonstrate that electrochemical cycling produces a near-surface Pd + Ge intermetallic with corrosion resistance far exceeding that of bulk PdGe. Corrosion resistance is enhanced by the presence of more cationic Ge at the surface, which acts as an intermetallic passivation layer. This enhanced corrosion resistance enables the material to be utilized as an electrocatalyst under conditions where bulk PdGe rapidly corrodes. The material exhibits exceptional activity for O_2_ reduction, which is enhanced by roughly an order of magnitude compared to Pd on a per Pd surface atom basis. Density functional theory suggests that activity promotion occurs via electronic and bifunctional effects. This work demonstrates that electrochemical near-surface alloying can produce remarkably corrosion resistant materials with superior electrocatalytic activity despite a reduced precious metals content.

## Graphical abstract

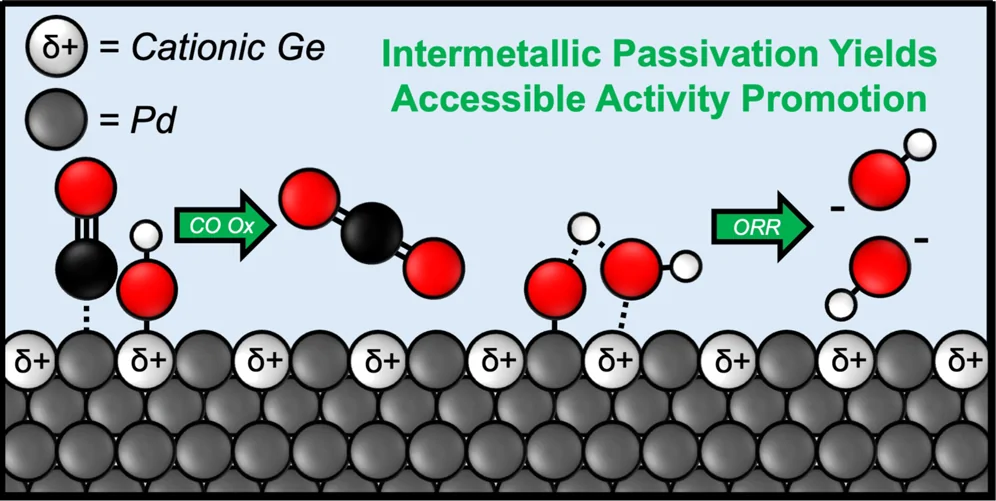

## Introduction

Electrocatalysis enables direct transformations between electrical and chemical energy, providing a path toward reducing the carbon footprint of fuel and chemical production.^1–8^ Unfortunately, the efficacy of electrocatalytic technologies is limited by a reliance on precious metal electrocatalysts that exhibit relatively low intrinsic activity.^9^ Thus, new electrocatalysts with both a reduced precious metals content and superior intrinsic activity are needed. Intermetallics, alloys consisting of electronically dissimilar metals,^10–13^ are a promising material platform for electrocatalyst development that could address these needs.^14–17^ The electronic dissimilarity of the constituent metals results in the formation of strong heteronuclear bonds that yield emergent properties ideal for catalytic applications.^18^ For example, strong heteronuclear bonding significantly modifies the electronic properties and surface reactivity of the constituent metals.^19,20^ Furthermore, this bonding imparts bifunctionality through the colocalization of active sites with vastly different reactivity. Unfortunately, intermetallics are susceptible to oxidative segregation in air and rapid corrosion under typical electrocatalytic conditions.^19,21–23^

The susceptibility of intermetallics to oxidative segregation in air and corrosion under electrochemical conditions has significantly hinderedtheir utilization as electrocatalysts.^24^ For example, propene partial oxidation over Pd could be promoted by Zn, Sn, and Ag due to their relatively high oxophilicity and relatively low carbophilicity.^25^ However, all of these metals rapidly corrode under the conditions of propene partial oxidation in acidic media.^25^ A variety of intermetallic synthesis strategies have been developed in an attempt to control the composition of the resulting material, improve stability, and limit corrosion. The most common approach is thermal annealing in an inert atmosphere to produce thermodynamically stable phases.^26^ However, producing phase-pure materials using this approach is nontrivial. Physical vapor deposition enables precise control over composition, morphology, and thickness but the resulting materials undergo significant changes upon air exposure.^26^ For example, a Ni–Al intermetallic catalyst consisting of Ni_2_Al_3_ and NiAl_3_ phases exhibited rapid corrosion during water electrolysis in alkaline media.^26^ Electrodeposition is another technique that is used to prepare intermetallics.^26^ However, preparing intermetallics via electrodeposition without subsequent annealing remains challenging and very few examples exist.^26^ Furthermore, these examples lack a complete understanding of the relationship between structure and electrocatalytic performance. Overall, the trade-off between the desirable properties exhibited by intermetallics and their susceptibility to corrosion under reaction conditions motivates the need for new synthesis strategies to prepare corrosion resistant intermetallics.

Herein, we demonstrate that cycling the potential applied to a Pd electrode in an electrolyte containing Ge cations generates a near-surface Pd + Ge intermetallic alloy. The resulting near-surface alloy exhibits no evidence of Ge corrosion upon anodic polarization in alkaline media, in stark contrast to the rapid Ge corrosion observed over bulk PdGe under identical conditions. In fact, the Ge corrosion resistance observed over the electrochemically fabricated intermetallic far exceeds that predicted by any existing thermodynamic model. The Ge initially incorporated into the Pd surface is highly cationic, enabling it to act as an intermetallic passivation layer that prevents the corrosion of the underlying material. This corrosion resistance enables the material to be utilized as an electrocatalyst for reactions that occur under conditions where bulk intermetallics rapidly corrode. Both CO oxidation and O_2_ reduction (ORR) in alkaline media are significantly promoted by near-surface alloying, with the latter enhanced by an order of magnitude on a per Pd surface atom basis. Density functional theory (DFT) calculations suggest that this activity promotion is the result of both electronic and bifunctional effects.

## Results

Bulk Pd–Ge intermetallic alloying only marginally improves the corrosion resistance of Ge. The impact of intermetallic alloying on the Ge corrosion potential was measured by performing cyclic voltammetry over Ge and PdGe in 0.1 M KOH using a custom electrolyte recirculation reactor that enables electrolyte purging under potential control (Supporting Information Figure S1). An irreversible oxidation wave was observed over both electrodes, as shown in Figure 1A. The charges of the observed oxidation waves are equivalent to the charges required to completely strip Ge from both electrodes. Interestingly, the onset potential of Ge corrosion is shifted positive by 345 mV over PdGe relative to pure Ge, demonstrating that bulk intermetallic alloying imparts Ge corrosion resistance into the material. Unfortunately, Ge corrosion from PdGe occurs well before the onset potential of either CO oxidation or ORR over pure Pd. Thus, the corrosion resistance imparted by bulk intermetallic alloying is insufficient to enable the material to be utilized as an electrocatalyst for either of these reactions.

**Figure 1:**
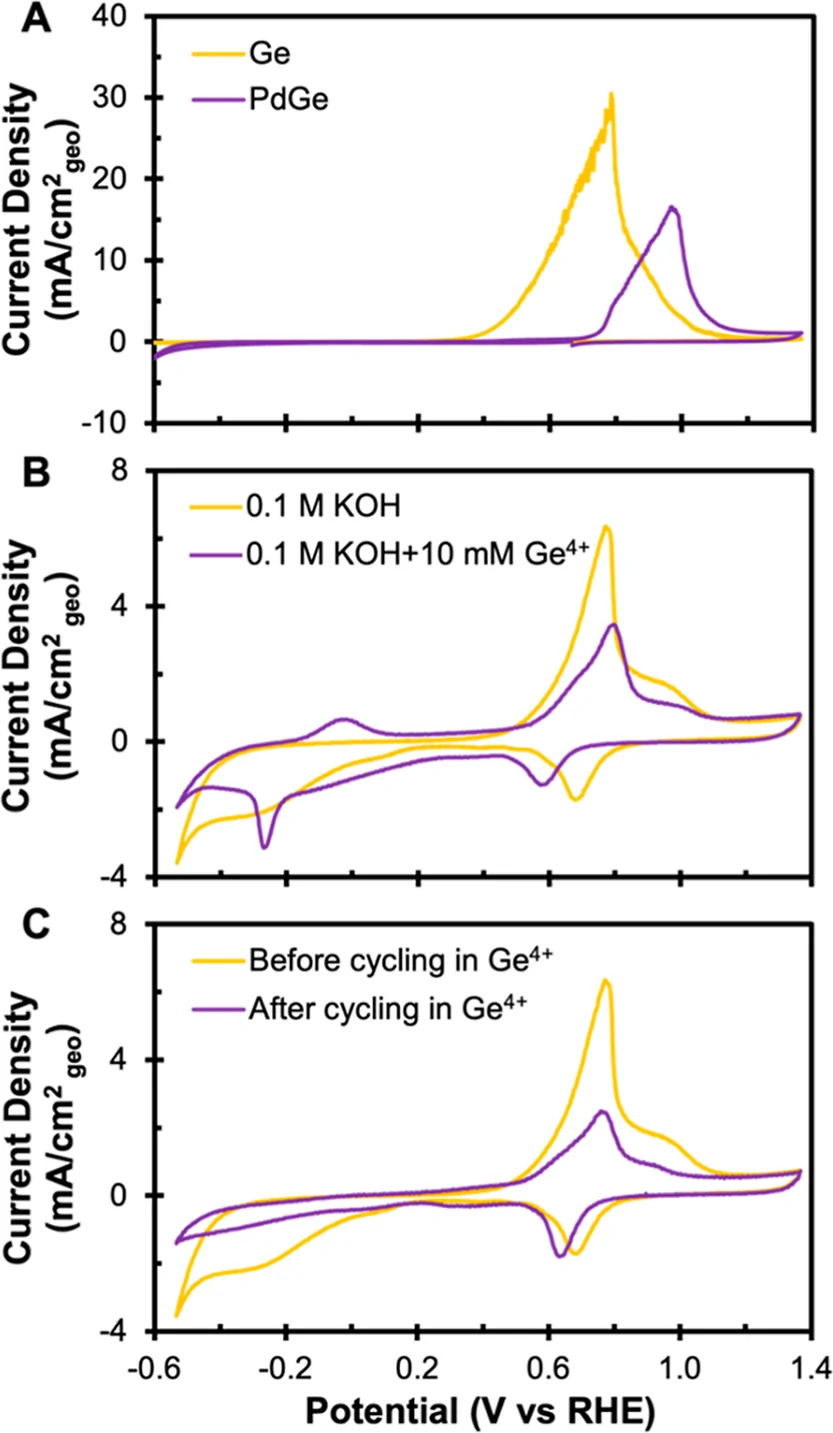
(A) Ge corrosion during cyclic voltammetry over Ge and PdGe in 0.1 M KOH. (B) Cyclic voltammetry over Pd in 0.1 M KOH with and without 10 mM Ge^4+^. (C) Cyclic voltammetry over Pd in 0.1 M KOH before and after performing 100 cycles in 0.1 M KOH+10 mM Ge^4+^.

Cycling the potential applied to a Pd electrode in an electrolyte containing Ge cations results in irreversible changes that indicate near-surface alloying has occur. The CV observed over Pd in 0.1 M KOH exhibits 3 main features: the reductive formation of PdH (−0.2 V vs RHE), the oxidative decomposition of PdH (+0.6 V vs RHE), and the reduction of Pd^δ+^ to Pd^0^ (+0.8 V vs RHE). After Ge^4+^ is added into the electrolyte, the CV changes over the course of ∼20 cycles before a steady state CV is obtained (Supporting Information Figure S2). The steady state CV exhibits redox waves associated with Ge underpotential deposition (UPD) and stripping at roughly −0.2 V vs RHE, shown in Figure 1B. The Ge underpotential deposition wave charge (∼0.5 mC/cm_geo_^2^) is significantly higher than the corresponding Ge underpotential stripping wave charge (∼0.4 mC/cm_geo_^2^), which suggests that some of the Ge deposited onto the Pd surface is irreversibly incorporated into the Pd surface during each cycle. The charge of the PdH decomposition wave is also suppressed, presumably because the irreversibly incorporated Ge hinders H diffusion into the Pd surface. The CVs were paused at +1.4 V vs RHE while the electrolyte was replaced with fresh 0.1 M KOH and then the CVs were resumed. Unsurprisingly, the Ge underpotential deposition and stripping waves were eliminated upon removal of Ge^4+^ from the electrolyte, as shown in Figure 1C. However, all other changes induced by cycling in the presence of Ge^4+^ persisted, corroborating the notion that cycling in the presence of Ge^4+^ results in irreversible Ge surface incorporation.

Electrochemical quartz crystal microbalance (EQCM) measurements suggest that the Ge initially incorporated into the Pd surface is highly cationic, requiring far fewer than 4 mol e^–^/mol Ge^4+^ to be electrodeposited. EQCM was used to monitor the rate of near-surface alloying during CV as a function of cycle number. The charge of the Ge underpotential deposition wave gradually increased with cycle number while the charge of the corresponding stripping wave remained constant, as shown in Figure 2A. The net mass deposited during each cycle was measured at the anodic vertex potential (+1.4 V vs RHE), which is significantly positive of the potentials where Ge underpotential deposition and stripping occur. Considerable mass was gained during each cycle (∼20 ng/[cm_geo_^2^*cycle]), as shown in Figure 2B. This mass gain was not observed in the absence of Ge^4+^ (Supporting Information Figure S3). Interestingly, the mass gained per cycle was constant despite the growing difference between the charges of the Ge underpotential deposition and stripping waves with cycle number. This suggests that the number of electrons transferred per irreversibly incorporated Ge atom increases with cycle number. The average number of electrons transferred per irreversibly incorporated Ge atom was calculated as a function of cycle number using eq 1

1 $$n_{incorporated\;Ge}=\frac{\left(Q_{GeUPD,reduction}-Q_{GeUPD,oxidation}\right){MW}_{Ge}}{\Delta m_{anodic\;vertex}F}$$

where *Q*_GeUPD_ is the charge of the Ge UPD wave, MW_Ge_ is the molecular weight of Ge, Δ*m*_anodicvertex_ is the mass change at the anodic vertex potential, and *F* is faraday’s constant. This analysis suggests that Ge incorporation initially requires only 2 mol e^–^/mol Ge^4+^, as shown in Figure 2C. The number of electrons transferred per incorporated Ge atom gradually increases with cycle number until the expected value of 4 mol e^–^/mol Ge^4+^ is reached at approximately 40 cycles. The cumulative mass gained by performing 40 cycles is equivalent to roughly a monolayer of Ge (Supporting Information calculation 1). Several Bragg reflections characteristic of bulk Pd_*x*_Ge_1–*x*_ intermetallic phases were observed via grazing incidence X-ray diffraction (GIXRD) after prolonged electrochemical cycling in the presence of Ge^4+^ (Supporting Information Figure S4). Unfortunately, the phase produced by near-surface alloying could not be definitely identified since the observed Bragg reflections are all characteristic of Pd_25_Ge_9_ (26.5 at. % Ge), Pd_21_Ge_8_ (27.6 at. % Ge), and Pd_2_Ge (33.3 at. % Ge).

**Figure 2:**
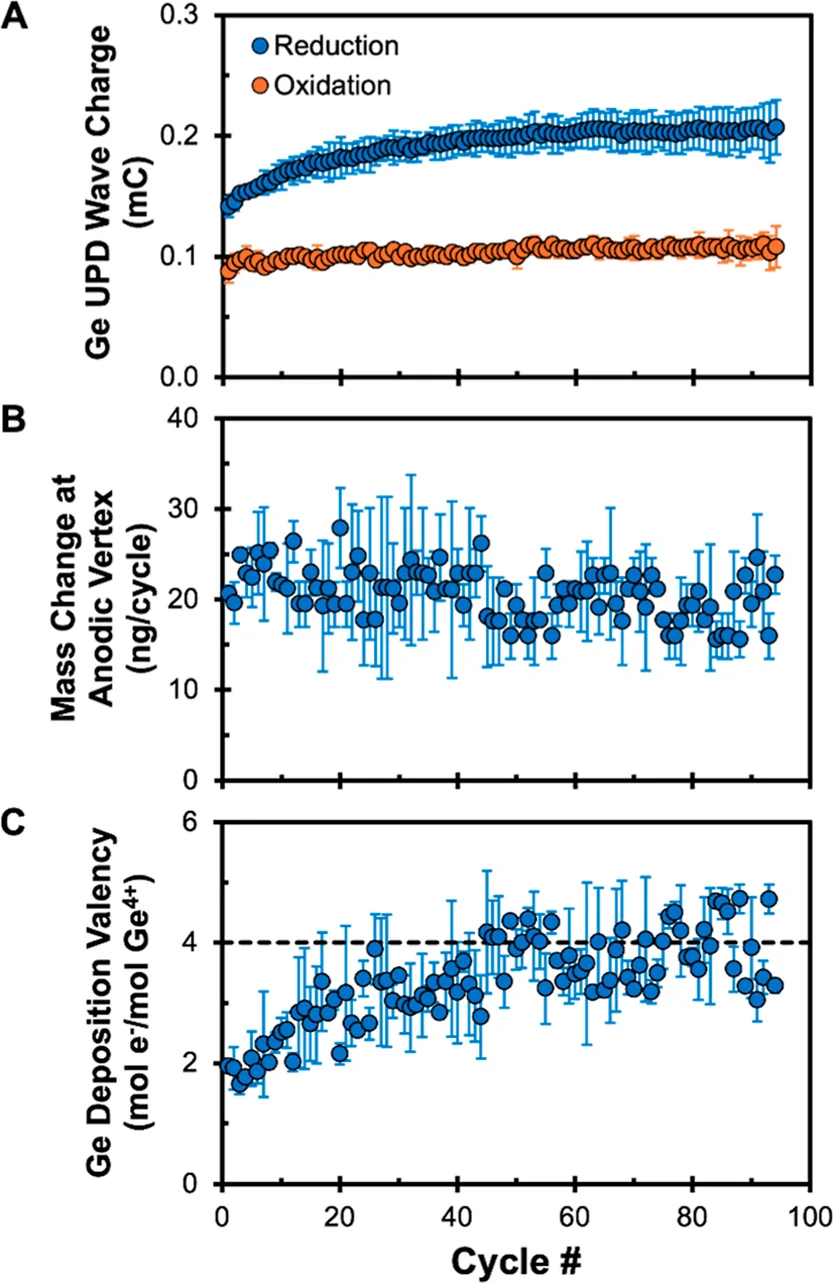
Electrochemical quartz crystal microbalance measurements of Ge near-surface alloying during cyclic voltammetry over Pd in 0.1 M KOH + 10 mM Ge^4+^ from −0.5 V to +1.4 V vs RHE: (A) Ge underpotential deposition and stripping wave charges vs cycle number. (B) Irreversible mass change observed at +1.4 V vs RHE vs cycle number. (C) Calculated number of electrons transferred per irreversibly incorporated Ge atom vs cycle number. Error bars calculated by taking the standard deviation of duplicate measurements.

X-ray photoelectron spectroscopy (XPS) measurements confirm that Ge is irreversibly incorporated into the Pd surface during CV. XPS measurements were performed over a series of Pd electrodes. The first electrode was immersed in the electrolyte containing Ge^4+^ at open circuit. This control sample exhibited a metallic Pd 3d binding energy and no Ge 3d signal, as shown in Figure 3A. The second sample was cycled 100 times in the presence of Ge^4+^ before terminating the CV at −0.4 V vs RHE, which is cathodic of the Ge UPD wave. This sample exhibited an elevated Pd 3d binding energy and contained both near-metallic Ge (roughly 1+) and oxidized Ge (4+) with a total near-surface Ge content of ∼26 at. %. The third sample was cycled in the same way, but the CV was terminated at +1.4 V vs RHE, which is significantly anodic of the Ge UPD stripping wave. This sample also exhibited an elevated Pd 3d binding energy and exclusively contained near-metallic Ge (roughly 1+) with a near-surface Ge content of ∼23 at. %. These observations suggest that there are 2 distinct types of Ge deposited during cycling. The first type is the Ge deposited onto the Pd surface, which oxidizes in air to GeO_2_ and is electrochemically stripped at potentials positive of roughly −0.2 V vs RHE. The second type is the Ge that is irreversibly incorporated into the Pd surface, which remains near-metallic upon air exposure and cannot be electrochemically stripped even by applying potentials of up to +1.4 V vs RHE.

**Figure 3:**
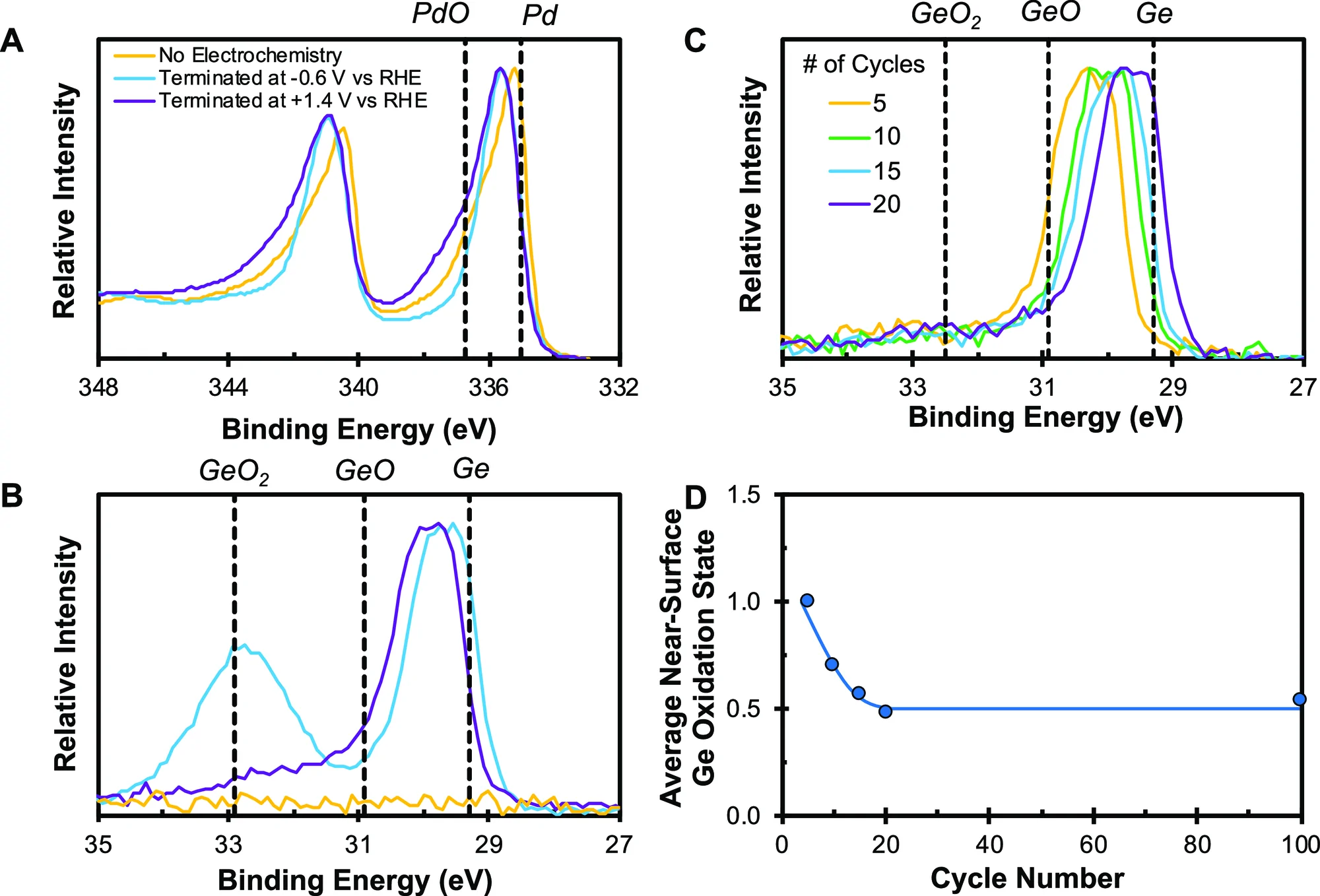
XPS spectra observed after performing 100 Ge incorporation cycles: (A) Pd 3d and (B) Ge 3d. (C) Ge 3d spectra and (D) near-surface Ge oxidation state as a function of the number of Ge incorporation cycles performed. Line added to guide the eye.

XPS measurements also reveal that the Ge incorporated into the Pd surface is more cationic than the Ge that is incorporated into the subsurface region. XPS measurements of the Ge 3d region were performed as a function of the number of Ge incorporation cycles. Ge incorporation cycling was terminated at +1.4 V vs RHE to ensure that only the irreversibly incorporated Ge remained in the near-surface region during subsequent XPS analysis. The Ge 3d photoelectron binding energy decreased rapidly with cycle number, as shown in Figure 3B. The observed spectra were used to calculate the average oxidation state of the near-surface Ge. The results suggest that the near-surface Ge becomes less cationic on average as more Ge is incorporated into the near-surface region, as shown in Figure 3C. Thus, these results qualitatively agree with the EQCM analysis above.

*Operando* electrochemical X-ray absorption near-edge structure (XANES) measurements confirm that the Ge irreversibly incorporated into the Pd surface exhibits exceptional corrosion resistance compared to either bulk Ge or bulk PdGe. The Pd + Ge near-surface alloy exhibits a lower Ge K-edge energy than GeO_2_, as shown in Figure 4. This suggests that the irreversibly incorporated Ge is in a reduced state. This conclusion is supported by the reduced Ge K-edge white line intensity, which typically scales with positive charge. Unfortunately, the lack of reliable Ge standards makes it difficult to quantify the Ge oxidation state present in the sample. During these experiments, the catalyst was held at each potential for approximately 1 h, resulting in a total operation time of roughly 10 h. Neither the Ge K-edge energy nor signal intensity significantly changed during this time. Thus, these measurements suggest that near-surface alloying increases the corrosion resistance of Ge by at least 1.4 V relative to pure Ge, enabling stability during prolonged operation under corrosive conditions. This is remarkable given that bulk intermetallic alloying only increases the Ge corrosion resistance by 345 mV. *Operando* electrochemical EXAFS of the Ge K-edge was also performed but the signal was insufficient to obtain structural information (Supporting Information Figure S5).

**Figure 4:**
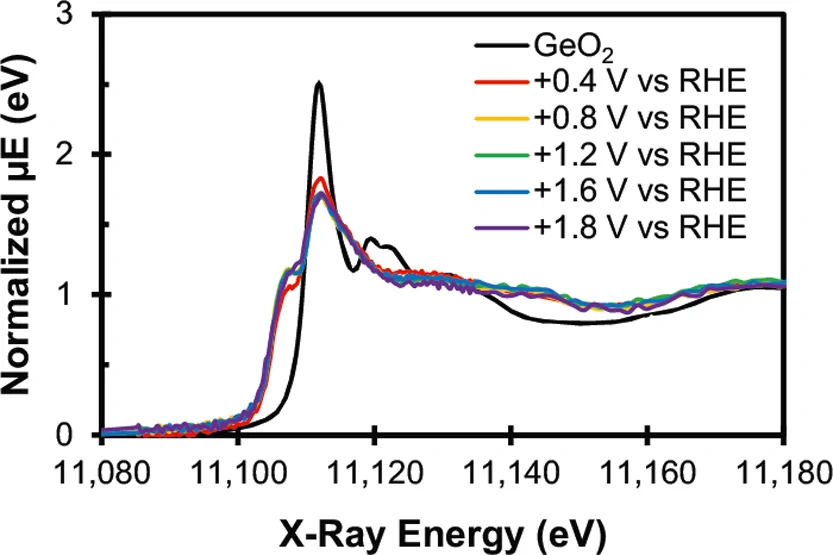
Operando electrochemical X-ray absorption near-edge structure of the Ge K-edge of Pd + Ge during chronoamperometry in 0.1 M KOH as a function of the applied potential.

The Pd + Ge near-surface intermetallic is enriched in Ge at the surface. CO stripping was performed before and after performing 100 Ge incorporation cycles to measure the surface composition present after near-surface alloying has occurred. After Ge incorporation cycling was complete, the potential was held at +1.4 V vs RHE while the electrolyte was replaced with fresh 0.1 M KOH. CO stripping was subsequently performed. The CO stripping charge was reduced by roughly a factor of 2 after near-surface alloying, as shown in Figure 5A. Low energy Ne ion scattering (LEIS) depth profiling was then performed to measure the near-surface composition gradient present in the material. The LEIS spectra exhibit inelastic scattering peaks at kinetic energies of roughly 1750 and 2400 eV, which correspond to Ge and Pd, respectively. Quantification of the observed spectra indicate that the 2 topmost atomic layers consist of ∼50 at. % Ge, whereas the subsurface layers consist of ∼27 at. % Ge, as shown in Figure 5B. Thus, both measurements suggest that the composition of the Pd + Ge near-surface alloy is ∼50 at. % Ge at the surface (Supporting Information calculation 2).

**Figure 5:**
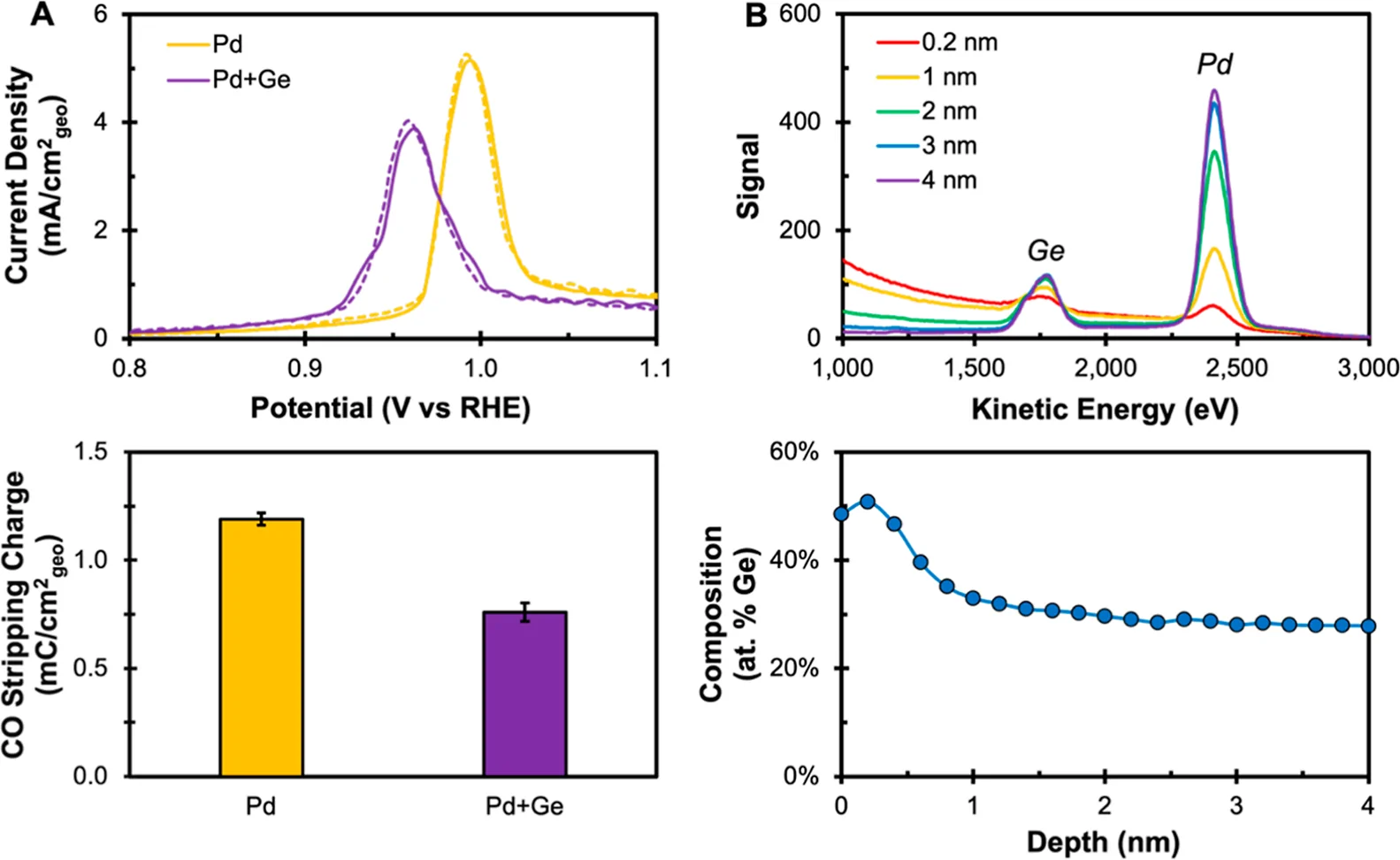
(A) Electrochemical CO stripping over Pd and Pd + Ge in 0.1 M KOH. Dotted line depicts results of repeat measurements. Error bars calculated by taking the standard deviation of triplicate measurements. (B) Quantitative low energy Ne-ion scattering depth profile of Pd + Ge. Line added to guide the eye.

DFT calculations suggest that near-surface alloying and Ge surface enrichment are both enthalpically driven by strong heteronuclear bonding. The DFT energy of exchanging a surface Ge atom with a subsurface Pd atom was calculated as a function of the surface composition. These calculations were performed for compositions of 0 and 33 at. % Ge in the sub-surface, which represent the initial and final sub-surface compositions present during electrochemical near-surface alloying. The energy of swapping a surface Ge atom with a subsurface Pd atom is exergonic for surface compositions >33 at. % Ge during the initial moments of alloying (for bulk compositions of 0 at. % Ge), as shown in Figure 6. This phenomenon is driven by strong heteronuclear bonding. Surface Ge atoms will swap with subsurface Pd atoms whenever doing so increases the number of Pd–Ge bonds. Thus, swapping is not favored when the surface Ge composition is low since there are already a large number of Pd–Ge bonds formed at the surface. However, once the surface composition rises above 33 at. % Ge, Pd–Ge bonding is increased by swapping a surface Ge atom with a subsurface Pd atom. However, the surface composition at which this swapping becomes exergonic also depends on the bulk composition. As the bulk Ge composition increases, higher Ge surface compositions are required to induce swapping. Interestingly, swapping only becomes exergonic for surface compositions >50 at. % Ge once the bulk composition has increased to 33 at. % Ge. Thus, higher Ge surface compositions are required to induce swapping once Ge has accumulated in the subsurface region. Thus, these calculations explain why electrochemical alloying produces a near-surface intermetallic enriched in Ge at the surface, as observed experimentally.

**Figure 6:**
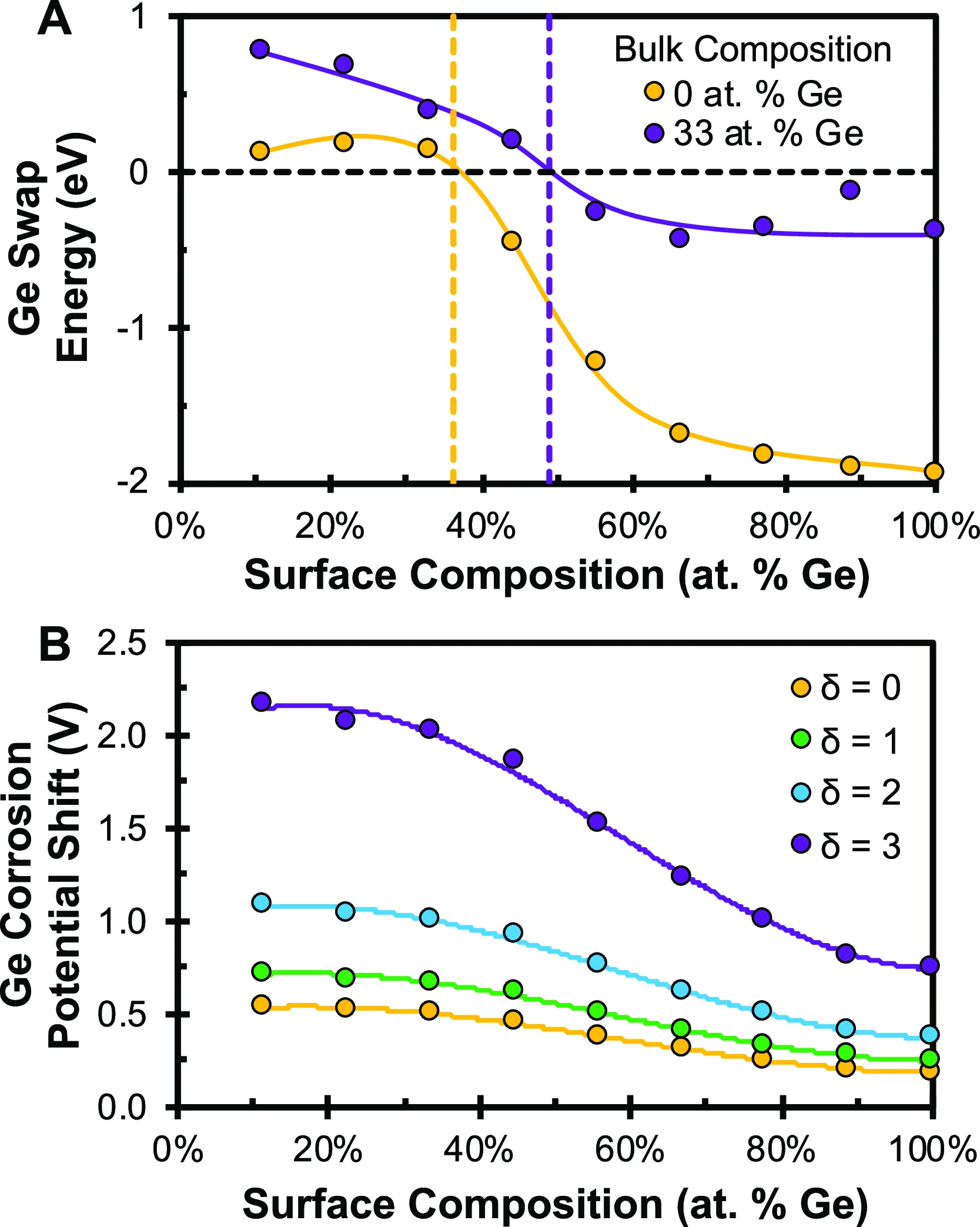
(A) Energy associated with exchanging a surface Ge atom with a subsurface Pd atom as a function of the surface composition for bulk compositions of 0 and 33 at. % Ge. (B) Ge corrosion resistance imparted by surface alloying with Pd(111) as a function of the number of electrons transferred per incorporated Ge atom upon corrosion. Lines added to guide the eye.

Intermetallic alloying imparts Ge corrosion resistance through three distinct mechanisms: intermetallic bonding, surface energetics, and increasing the oxidation state of the near-surface Ge. The magnitude of the corrosion resistance imparted by bulk intermetallic alloying can be calculated using eq 2

2 $$\Delta E_{{Ge}^{4+}/{Ge}^{\delta +}}^{0}=\frac{-\left[{\Delta H}_{f,{Pd}_{x}{Ge}_{1-x}}+\left(\gamma_{{Pd}_{x}{Ge}_{1-x}}-\gamma_{Pd}\right)\right]}{x_{Ge}\left(4-\delta \right)F}$$

where Δ*E*_Ge^4+^/Ge^δ+^_ is the change is the Ge standard reduction potential, Δ*H*_*f,*PdxGe1–_*x*__ is the formation energy of the bulk intermetallic, γ_PdxGe_1–*x*__ is the surface energy of the bulk intermetallic, *x*_Ge_ is the bulk Ge composition, δ is the oxidation state of the Ge present in the intermetallic, and *F* is faraday’s constant. For bulk PdGe, the surface composition was assumed to be equivalent to the bulk and the surface energy was assumed to be a linear combination of the surface energies of Pd and Ge. Using this approach, the Ge corrosion potential from bulk PdGe was estimated to be roughly 330 mV positive of the corrosion potential of bulk Ge. This is in excellent agreement with our experimental measurement of 345 mV. Thus, this equation can be used to estimate the magnitude of the Ge corrosion resistance imparted by near-surface intermetallic alloying. The Ge corrosion potential from Pd(111) + Ge was calculated as a function of the surface composition and number of electrons transferred per Ge atom upon corrosion. The Ge corrosion potential increases as both the Ge surface composition and number of electrons transferred per Ge surface atom decrease, as shown in Figure 6B. Ge corrosion resistance is enhanced by roughly 1 V for surface compositions of 50 at. % Ge present in the 2+ oxidation state, which is in close agreement with the magnitude of the corrosion resistance observed via XPS (Figure 3) and XAS (Figure 4). Thus, the cationic Ge present at the surface acts as an intermetallic passivation layer that prevents the corrosion of the underlying Ge despite its thermodynamic instability.

The enhanced corrosion resistance afforded by near-surface intermetallic alloying enables utilization as an electrocatalyst for reactions that occur anodic of the corrosion potential of bulk PdGe. The impact of Ge incorporation on the electrocatalytic activity of Pd for both CO oxidation and ORR were investigated in alkaline media. The potential required to obtain +1 mA/cm^2^ of CO oxidation current was reduced by ∼150 mV upon Ge near-surface alloying, as shown in Figure 7A. CO oxidation over Pd + Ge occurs anodic of the bulk PdGe corrosion potential. Thus, this activity promotion is only accessible due to the enhanced corrosion resistance afforded by the electrochemical near-surface alloying process described herein. The rate-determining step of CO oxidation is the nonelectrochemical coupling of CO* (where “*” denotes a surface adsorbed species) and OH* to form COOH*.^27,28^ The binding energies of CO* and OH* were calculated relative to Pd(111) across various Pd(111) + Ge and bulk Pd_*x*_Ge_1–*x*_ intermetallic surfaces. Ge incorporation weakens CO* binding and increases OH* binding relative to Pd(111) across the various surfaces investigated, as shown in Figure 7B. The binding energies calculated in all models are reported in the Supporting Information (Table S1). The weakening of the CO* binding energy is due to both electronic and geometric effects. Ge incorporation shifts the Pd d-band away from the Fermi level toward higher binding energies, which reduces the CO* binding energy by reducing electron back-donation.^29^ However, the incorporation of Ge also weakens the CO* binding energy by altering the preferred adsorption site. CO* preferentially binds at the 3-fold hollow site over Pd(111), as shown in Figure 7C. However, Ge incorporation eliminates these surface sites. As a result, CO* must bind at Pd–Pd bridge sites or Pd atop sites on the various surfaces investigated. The reduced coordination of these binding sites yields a weaker CO* binding energy. The activation barrier of coupling CO* and OH* to form COOH* scales inversely with the CO* adsorption energy.^28^ As a result, Ge incorporation promotes CO oxidation by weakening the CO* adsorption energy, which reduces the barrier for COOH* formation. The increased OH* binding energy is predominately caused by a change in the preferred adsorption site. OH* also preferentially binds at the 3-fold hollow site over Pd(111), meaning CO* and OH* are typically in direct competition for surface active sites. The incorporation of Ge into the Pd surface changes the preferred OH* adsorption site to the Ge atop site. Thus, the incorporation of Ge into the Pd surface creates unique adsorption sites for each reactant that is involved in the rate-determining step of CO oxidation. Ge promotes CO oxidation over Pd by a similar mechanism as Ru promotes CO oxidation over Pt.^30–32^ However, the Pt–Ru bond is relatively weak and is insufficient to establish long-range structural order. As a result, Pt–Ru exhibits a wide distribution of surface active sites with different CO oxidation activities, which yields a broad CO oxidation wave.^17^ Some of the sites in PtRu are more active than pure Pt while others are less active. Conversely, the Pd–Ge bond is exceptionally strong and results in long-range structural order. This long-range order yields a narrow distribution of surface active sites and a concomitantly narrow CO oxidation wave.

**Figure 7:**
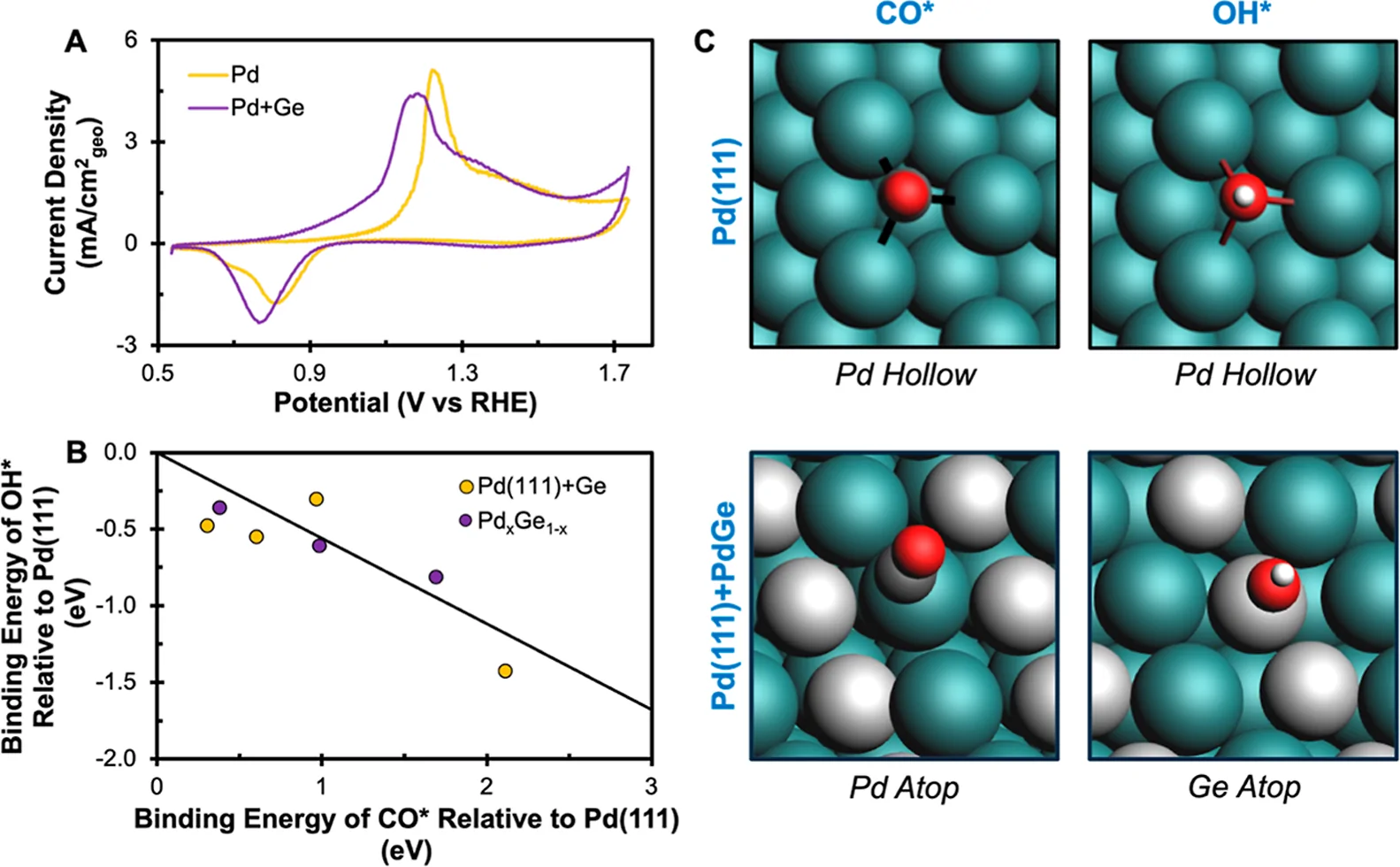
(A) CO oxidation over Pd and Pd + Ge in 0.1 M KOH. (B) Correlation between the binding energies of CO* and OH* over Pd(111) + Ge and Pd_*x*_Ge_*x*–1_ intermetallic alloys relative to Pd(111). Solid black line represents a linear fit. (C) Preferred adsorption sites of CO* and OH* over Pd(111) and Pd_25_Ge_9_(111).

Pd is the most active monometallic electrocatalyst for ORR in alkaline conditions.^33,34^ Interestingly, Ge near-surface alloying shifts the half-wave potential of ORR positively by roughly 40 mV compared to pure Pd, as shown in Figure 8A. As a result, the Pd-normalized ORR activity is enhanced by an order of magnitude by Ge near-surface alloying, as shown in Figure 8B. The potential used to make this comparison was carefully selected such that the current densities were <10% of the limiting value over both materials, which ensures that the comparison reflects intrinsic activity differences. Interestingly, Ge incorporation slightly increases the ORR limiting current density, which suggests that Ge incorporation increases the selectivity for H_2_O at the expense of H_2_O_2_. The rate-determining step of ORR over Pd is hypothesized to be the reduction of OH* to H_2_O.^35,36^ Thus, weakening the OH* adsorption energy should elevate the ORR activity of Pd. While Ge surface alloying introduces strong OH* binding sites, it also weakens the OH* adsorption energy to the remaining Pd surface sites, as shown in Figure 8C (Supporting Information Figure S7). These binding energy shifts are insufficient to alter the rate-determining step of the reaction. This could explain the origins of the ORR activity promotion obtained from near-surface intermetallic alloying.

**Figure 8:**
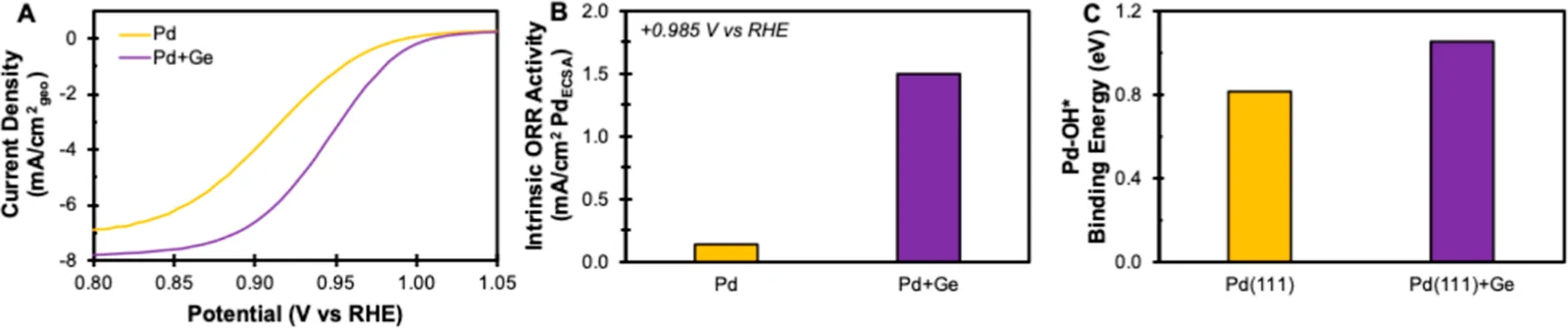
(A) Oxygen reduction over Pd and Pd + Ge in 0.1 M KOH during the positive going scan. (B) Comparison of the surface Pd-normalized ORR activity of Pd and Pd + Ge at +0.985 V vs RHE. (C) Comparison of the Pd–OH* binding energy over Pd(111) and Pd_25_Ge_9_(111).

## Conclusion

Intermetallics are a promising platform for electrocatalyst development, but their efficacy has been limited by their susceptibility to corrosion. Ge underpotential deposition over Pd results in irreversible near-surface alloying. This alloying is enthalpically driven, with deposited Ge spontaneously entering the Pd surface in order to maximize the number of heteronuclear bonds. The Ge incorporated into the Pd surface is more cationic than the Ge incorporated into the subsurface region. The cationic nature of the Ge present at the surface enables it to act as an intermetallic passivation layer, imparting significant corrosion resistance into the material. This exceptional corrosion resistance enables the material to be utilized as an electrocatalyst under conditions where no existing thermodynamic model would have predicted the material to be stable. The incorporation of Ge into the Pd surface promotes both CO oxidation and ORR while reducing the precious metal content of the surface. This activity promotion enhances the Pd-normalized ORR activity by an order of magnitude due to a combination of electronic and bifunctional effects. Thus, this work opens up an entire material class for electrocatalytic applications under conditions that were previously thought impossible. Furthermore, this work demonstrates that intermetallics are a viable material platform for reducing the precious metal content of electrocatalysts while simultaneously enhancing their intrinsic activity. Extending this synthesis protocol to nanoparticle-based materials could provide a versatile method for producing highly active electrocatalysts with a reduced precious metals content in a format amenable to complementary structural characterization.

## Supplementary Material



## Notes

The Supporting Information contains a detailed description of all experimental and computation methods, a schematic of the electrochemical flow cell, extended electrochemistry data, extended electrochemical quartz crystal microbalance data, equations used to calculate the monolayer equivalents of Ge deposited per cycle and the Ge surface composition, extended characterization data (grazing incidence X-ray diffraction and extended X-ray absorption fine structure), and a table summarizing the results of DFT calculations (PDF)
