# Supplementary material for: Intermetallic Passivation Imparts Corrosion Resistance and Enables Intrinsic Electrocatalytic Activity Promotion
Source: J Am Chem Soc. 2026 Sep 30;148(40):43081–8. doi: 10.1021/jacs.6c09853 (PMC13674080; doi:10.1021/jacs.6c09853)
Supplement: Supplementary file 1 [file ja6c09853_si_001.pdf]

Supplementary Information

Intermetallic Passivation Imparts Corrosion Resistance and Enables Intrinsic Electrocatalytic Activity Promotion

Majd M. Matalkeh[1], Jin Li[1], Andrew J. Wong[1], Alan Dang[1], Bryan D. Vogt[1], Michael J. Janik[1], Ezra L. Clark[1]

[1]The Pennsylvania State University, Robert V. Waltemeyer Department of Chemical Engineering, University Park, Pennsylvania 16802, USA

[*]Corresponding Author (ezra.lee.clark@psu.edu)

Experimental Methods

Thin Film Deposition

Pd thin films were deposited onto Si(100) (University Wafer) under Ar using a Kurt J. Lesker CMS-18 sputtering system. A 5 nm Ti (99.999%, Kurt J. Lesker) adhesion layer was deposited followed by a 100 nm Pd (99.999%, Kurt J. Lesker).

Electrochemical Reactor Setup

The electrochemical reactor setup consists of an electrochemical flow cell, a peristaltic pump (equipped with Masterflex Chem-Durance Bio tubing), electrolyte selection valves (Vici, ChemInert), and a series of electrolyte reservoirs. This configuration enables the electrolyte to be exchanged while holding the catalyst under potential control. The custom electrochemical flow cell was machined from polyether ether ketone (PEEK). The electrochemical cell contained working and counter electrode chambers with a parallel electrode geometry. The working and counter electrodes were circular with an exposed geometric area of 1 cm^2. The counter electrode was a glassy carbon plate (Alfa Aesar) and the reference electrode was an Ag/AgCl electrode (Pine) that was connected to the reactor using a luggin capillary. The working and counter electrode chambers were separated with an anion exchange membrane (Selemion AMVN AGC Inc). Electrochemistry was performed using a Biologic VSP-300 potentiostat. All electrochemical measurements were recorded versus the reference electrode and converted to the reversible hydrogen electrode (RHE) scale. All experiments were recorded at an electrolyte flow rate of 200 mL/min.

Electrochemical Near-Surface Alloying

The Pd thin films were cut into square electrodes (2.5x2.5 cm^2) and cycled in 0.1 M KOH (99.995%, Sigma Aldrich) containing 10 mM Ge^{4+} (GeO_2, >99.99%, Sigma Aldrich) from –0.6 to +1.4 V vs RHE at 200 mV/sec.

Electrochemical CO Oxidation and Stripping

CO oxidation was performed in 0.1 M KOH saturated with CO by cycling the applied potential from +0.6 to +1.8 V vs RHE at 200 mv/sec. CO stripping was performed by pumping electrolyte saturated with CO through the reactor, pumping fresh electrolyte saturated with Ar through the reactor, and then performing CVs from +0.6 to +1.2 V vs RHE.

Electrocatalytic Oxygen Reduction (ORR)

ORR was performed in 0.1 M KOH saturated with O_2 by cycling the potential from +0.7 to +1.1 V vs RHE at 200 mV/sec.

Electrochemical Quartz-Crystal Microbalance (EQCM)

Electrochemical quartz crystal microbalance measurements were performed using a Biolin Scientific QSense. The EQCM setup consists of the QEM 401 module, a peristaltic pump, and a series of electrolyte reservoirs. The working electrode was a commercial 5 MHz gold electrode that was sputter coated with 100 nm of Pd. A Ag/AgCl electrode and a Pt plate were used as the reference and counter electrodes, respectively. An electrolyte flowrate of 0.5 mL/min was used for all measurements. The mass change at the anodic vertex potential was calculated using the Sauerbrey equation:

$$\Delta m = -C\frac{\Delta f}{n} \quad (1)$$

where

Δm	Mass change (ng/cm^2)
C	Sensitivity constant (17.7 ng/cm^2 * Hz)
Δf	Frequency change (Hz)
n	Overtone (n = 5)

Low Energy Ne Ion Scattering (LEIS)

Ne-LEIS was performed using the Qtac 100 High Sensitivity-Low Energy Ion Scattering (HS-LEIS) spectrometer system. Spectra were measured using a beam energy of 5 keV, which was rastered over a 2.5x2.5 mm^2 area. The surface composition was quantified using Pd and Ge relative sensitivity factors measured over pure thin films.

X-Ray Photoelectron Spectroscopy (XPS)

XPS was performed using a PHI Versprobe II. XPS was performed using Al Kα radiation. Core level spectra were recorded using a pass energy of 27 eV and step size of 0.1 eV. Binding energy calibration was performed by setting the C 1s binding energy to 284.8 eV. Quantification was performed by integrating the observed signals with a Shirley background. Reference data was obtained from XPS Simplified.

X-Ray Absorption Spectroscopy (XAS)

XAS was performed at beamline 9–3 of the Stanford Synchrotron Radiation Light source at the SLAC National Accelerator Laboratory. Beamline 9–3 is a 16-pole, 2-Tesla wiggler side station with a Rh-coated vertically collimating mirror for harmonic rejection and a Rh-coated cylindrically bent mirror for focusing. The photon energy was selected using a liquid nitrogen cooled, double-crystal Si (220) $\varphi = 0°$ monochromator. The ionization chambers were filled with N_2 with a 1800 V negative polarity applied bias. *Operando* XAS measurements were collected in transmission mode using continuous scanning at a rate of 20 min per scan. A total of 2 scans were collected for signal averaging for each applied potential. Data processing (merging, normalization, and background subtraction) was performed using Athena software for the Demeter package. Electrochemical experiments were performed in a custom electrochemical flow cell machined from PEEK. The counter electrode was a glassy carbon plate (Alfa Aesar) and the reference electrode was a Ag/AgCl electrode (Innovative Instruments). Electrochemistry was performed using a Biologic VSP-200 potentiostat. All electrochemical measurements were recorded versus the reference electrode and converted to the reversible hydrogen electrode (RHE) scale.

Grazing Incidence X-ray Diffraction (GIXRD)

XRD was performed using Malvern Panalytical Empyrean III x-ray diffractometer using Cu Kα radiation (40 kV, 45 mA). The diffractometer was equipped with a parallel beam mirror, a 10 mm mask, and a $1/2^0$ soller slit. Grazing incidence diffraction was performed with an incident angle of 0.2^0. Phase identification was performed using reference data obtained from the International Center for Diffraction Data (ICDD) database.

Computational Methods

Electronic Structure Calculation

The Vienna Ab initio software (VASP), interfaced through the Atomic Simulation Environment (ASE), was used to perform the electronic structure calculations.[1–4] The projector augmented wave (PAW) method was used to approximate the core-valence electrons. The exchange and correlation energies of the electrons were calculated using the generalized gradient approximation (GGA) with the Perdew, Burke, and Ernzerhof (PBE) functional.[5–8] A cutoff energy of 450 eV was used to truncate the plane wave basis set. The convergence limits for the ionic and electronic sets used values of 0.05 eV/Å and 10^{-5} eV respectively. The Methfessel-Paxton scheme was used to approximate the surface model Fermi smearing width by using a value of 0.2 eV.[9] The semi-core d-states for Ge atoms are treated as explicit valence electron states. Both QVASP and VASPKit were used to assist in the analysis of partial density of states (PDOS) and d-band centers.

Graph Neural Networks

- The Gemnet-oc22 model's s2ef method was used for screening all the possible configurations
- Fmax=0.05 eV/Å

Model Construction

The Pd(111) surface model was represented 4x4 four-layer slab with a 15 Å vacuum, where bottom two layers are frozen to represent the bulk metal. Pd(111)+Ge alloys are modeled by substituting the previously mentioned Pd surface and/or subsurface atoms with Ge atoms to achieve a ½ or 1 ML coverage. Most stable orientation at a given coverage of Ge determined from surface energy calculations (see discussed below). The Monkhorst-Pack method was used to sample the Brillouin zone for periodic systems.[10] A 4x4x1 k-point grid was used for the Pd(111) and Pd(111)+Ge alloys.

Pd+Ge Intermetallic Surfaces

- PdGe bulk structures are obtained from the Materials Project to enumerate all low-index surfaces (h,l,k<=2) and various terminations. The minimum slab and vacuum thickness are both set to 10.0 Å.
- For surface energy calculations: Models are symmetric on both sides with atoms within 2 Å from both top and bottom surfaces fixed.
- For adsorption energy calculations: Only atoms 2 Å below the top surface are fixed to allow dynamic responses in the slab.

Calculating Surface Energies

Due to the non-identical stoichiometry between the surface slab and the bulk, we calculated the surface energies by subtracting the bulk energies of the constituent elements. For the Ge_xPd_y system, we used the bulk energies of Pd,

$$\frac{x}{a}{Ge_a Pd_b}^{bulk} + (y - \frac{bx}{a})Pd^{bulk} \rightarrow {Ge_x Pd_y}^{surface}$$

$$E^{surface} = \frac{E^{Ge_xPd_y{}^{surface}} - \frac{x}{a}E^{Ge_aPd_b{}^{bulk}} - (y - \frac{bx}{a})E^{Pd^{bulk}}}{2A}$$

where $E^{Ge_xPd_y{}^{surface}}$ represents DFT energy of the Ge_xPd_y surface, $E^{Ge_aPd_b{}^{bulk}}$ represents DFT energy of the Ge_aPd_b bulk, $E^{Pd^{bulk}}$ represents DFT energy of the Pd bulk, A represents the surface area of the surface model.

Ge Swap Energies:

A specific swap of a surface Ge atom with a subsurface Pd atom into a 5-layer, 3x3 Pd (111) surface model with different surface Ge compositions was performed. Ge Swap Energy can be determined by calculating the energy difference between the after and before Ge swap as:

$$E_{swap} = E_{after} - E_{before}$$

Where

E_{after} DFT energy of the system after the swap

E_{before} DFT energy of the system before the swap.

Equilibrium Potential of Surface Ge Oxidation

The oxidation resistance of Ge atoms of a surface model can be obtained from the equilibrium potential of the following reaction:

$$Ge_xPd_y^{surface} + Pd_{a-y}^{bulk} \rightarrow xGe^{4+} + Pd_a^{surface} + 4xe^-$$

It can be divided into the following three reactions based on thermal cycling:

$$Ge_xPd_y^{surface} + Pd_{a-y}^{bulk} \rightarrow xGe^{bulk} + Pd_a^{bulk}$$
$$xGe^{bulk} + Pd_a^{bulk} \rightarrow xGe^{bulk} + Pd_a^{surface}$$
$$xGe^{bulk} + Pd_a^{surface} \rightarrow xGe^{4+} + Pd_a^{surface} + 4xe^-$$

The relative equilibrium potential is as follows:

$$V = \frac{xE^{Ge^{bulk}} + E^{Pd_a^{surface}} - (a-y)E^{Pd^{bulk}} - E^{Ge_xPd_y^{surface}}}{4x}$$

where $E^{Ge^{bulk}}$ is the DFT energy of Ge bulk per Ge atom, $E^{Pd^{bulk}}$ is the DFT energy of Pd bulk per Pd atom, $E^{Pd_a^{surface}}$ is the DFT energy of a Pd(111) surface with total a atoms, $E^{Ge_xPd_y^{surface}}$ is the DFT energy of an intermetallic surface or surface alloy surface.

The oxidation resistance of Ge atoms of bulk intermetallic can be obtained from the equilibrium potential of the following reaction:

$$Ge_xPd_y^{bulk} \rightarrow xGe^{4+} + yPd^{bulk} + 4xe^-$$

It can be divided into the following two reactions based on thermal cycling:

$$Ge_xPd_y^{bulk} \rightarrow xGe^{bulk} + yPd^{bulk}$$
$$xGe^{bulk} + yPd^{bulk} \rightarrow xGe^{4+} + yPd^{bulk} + 4xe^-$$

The relative equilibrium potential is as follows:

$$V = \frac{xE^{Ge^{bulk}} + yE^{Pd^{bulk}} - E^{Ge_xPd_y^{bulk}}}{4x}$$

where $E^{Ge^{bulk}}$ is the DFT energy of Ge bulk per Ge atom, $E^{Pd^{bulk}}$ is the DFT energy of Pd bulk per Pd atom, $E^{Ge_xPd_y^{bulk}}$ is the DFT energy of an intermetallic bulk.

Binding Energies

The adsorption of CO^* on Pd + Ge surface alloys is determined from the following reaction as:

$$CO_{(g)} + \ * \rightarrow CO^* \ (\#)$$

where * is the bare Pd (+Ge alloy) Surface. The binding energy of CO^*, $E_{ads,CO*}$, is calculated in the form as:

$$E_{ads,CO*} = E_{CO*} - E_* - E_{CO_{(g)}} \ (\#)$$

where E_{CO*} is DFT energy of CO* adsorbed on the surface, E_* is the DFT energy of the bare metal surface, and $E_{CO_{(g)}}$ is the energy of gas-phase CO.

The OH* adsorption on Pd+Ge alloy surfaces is determined using the following reaction:

$$H_2O_{(g)} + \ * \rightarrow OH^* + \frac{1}{2}H_{2\,(g)} \ (\#)$$

The binding energy of OH*, $E_{ads,OH*}$, is calculated as:

$$E_{ads,OH*} = \frac{1}{2}E_{H_{2\,(g)}} + E_{OH^*} - E_{H_2O_{(g)}} \ (\#)$$

where DFT energy of hydrogen gas is $E_{H_{2\,(g)}}$, DFT energy of surface-bound hydroxyl is E_{OH^*}, and DFT energy of gas-phase water is $E_{H_2O_{(g)}}$.

References

1. Kresse, G. & Furthmiiller B ', J. *Efficiency of Ab-Initio Total Energy Calculations for Metals and Semiconductors Using a Plane-Wave Basis Set*. *Computational Materials Science* vol. 6 (1996).
2. Hjorth Larsen, A. *et al.* The atomic simulation environment - A Python library for working with atoms. *Journal of Physics Condensed Matter* vol. 29 Preprint at https://doi.org/10.1088/1361-648X/aa680e (2017).
3. Kresse, G. *Ab Initio Molecular Dynamics for Liquid Metals*. *Journal of Non-Crystalline Solids 192&* vol. 193 (1995).
4. Kresse, G. *Ab Initio Molecular-Dynamics Simulation of the Liquid-Metal-Amorphous-Semiconductor Transition in Germanium*. *PHYSICAL REVIEW* vol. 8.
5. Blochl, P. E. *Projector Augmented-+rave Method*. *PHYSICAL REVIEW B VOLUME* vol. 50.
6. Kresse, G. & Joubert, D. *From Ultrasoft Pseudopotentials to the Projector Augmented-Wave Method*.
7. Perdew, J. P. *et al. Atoms, Molecules, Solids, and Surfaces: Applications of the Generalized Gradient Approximation for Exchange and Correlation*. *PHYSICAL REVIEW B* vol. 46.
8. Perdew, J. P., Burke, K. & Ernzerhof, M. *Generalized Gradient Approximation Made Simple*. (1996).
9. Methfessel, M. & Paxton, A. T. *High-Precision Sampling for Brillouin-Zone Integration in Metals*. *PHYSICAL REVIEW B* vol. 40.

10. Monkhorst, H. J. & Pack, J. D. *Special Points for Brillonin-Zone Integrations**. *NUMBER* vol. 13 (1976).

Supplementary Fig. 1: Schematic of the Electrochemical Reactor Setup

A custom electrochemical reactor setup was designed and constructed. The setup consists of an electrochemical flow cell, a peristaltic pump, electrolyte selection valves, and a series of electrolyte reservoirs, as shown in Supplementary Fig. 1. This configuration enables the electrolyte to be exchanged while holding the electrocatalyst under potential control, which cathodically protects it from oxidation.

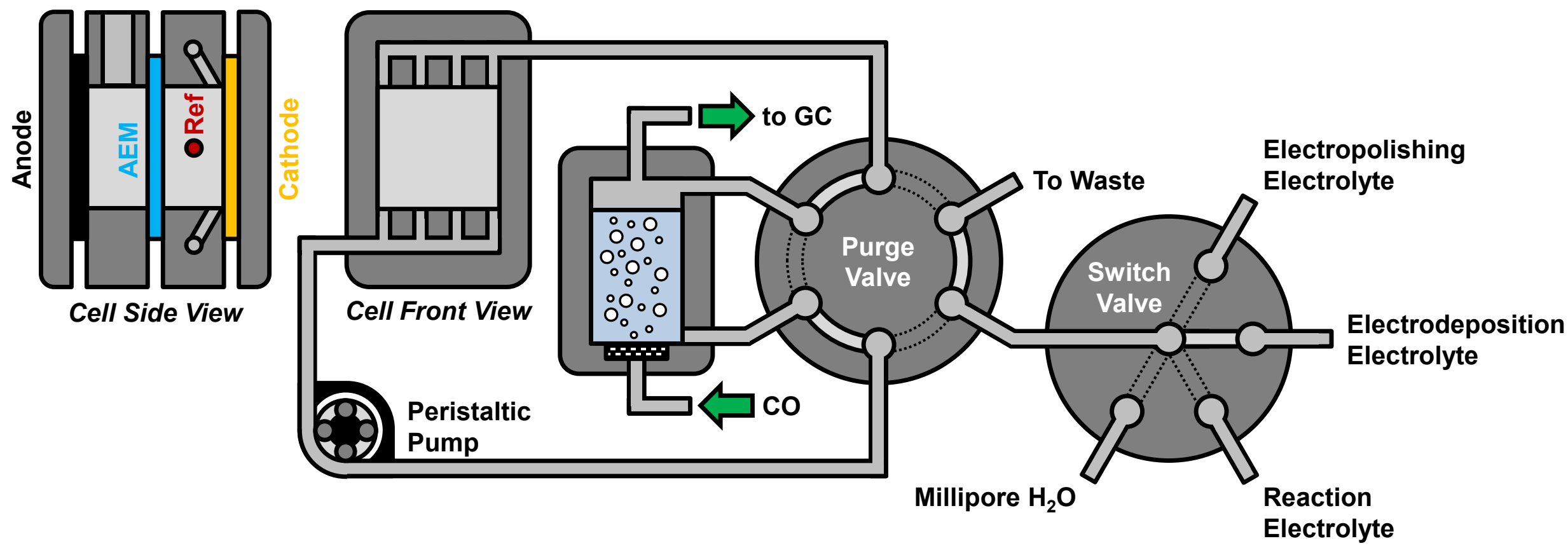

Supplementary Fig. 1 *– Schematic of the electrochemical reactor setup.*

Supplementary Fig. 2: Evolution of the Cyclic Voltammogram over Pd after Ge^{4+} is Added into the Electrolyte

After Ge^{4+} is added into the electrolyte, the CV changes over the course of ~20 cycles before a steady state CV is obtained, as shown in Supplementary Fig. 2. The Ge underpotential deposition peak appears after approximately 5 cycles. With continued cycling, the reduction peak intensity gradually increases and eventually reaches a steady state after approximately 20 cycles.

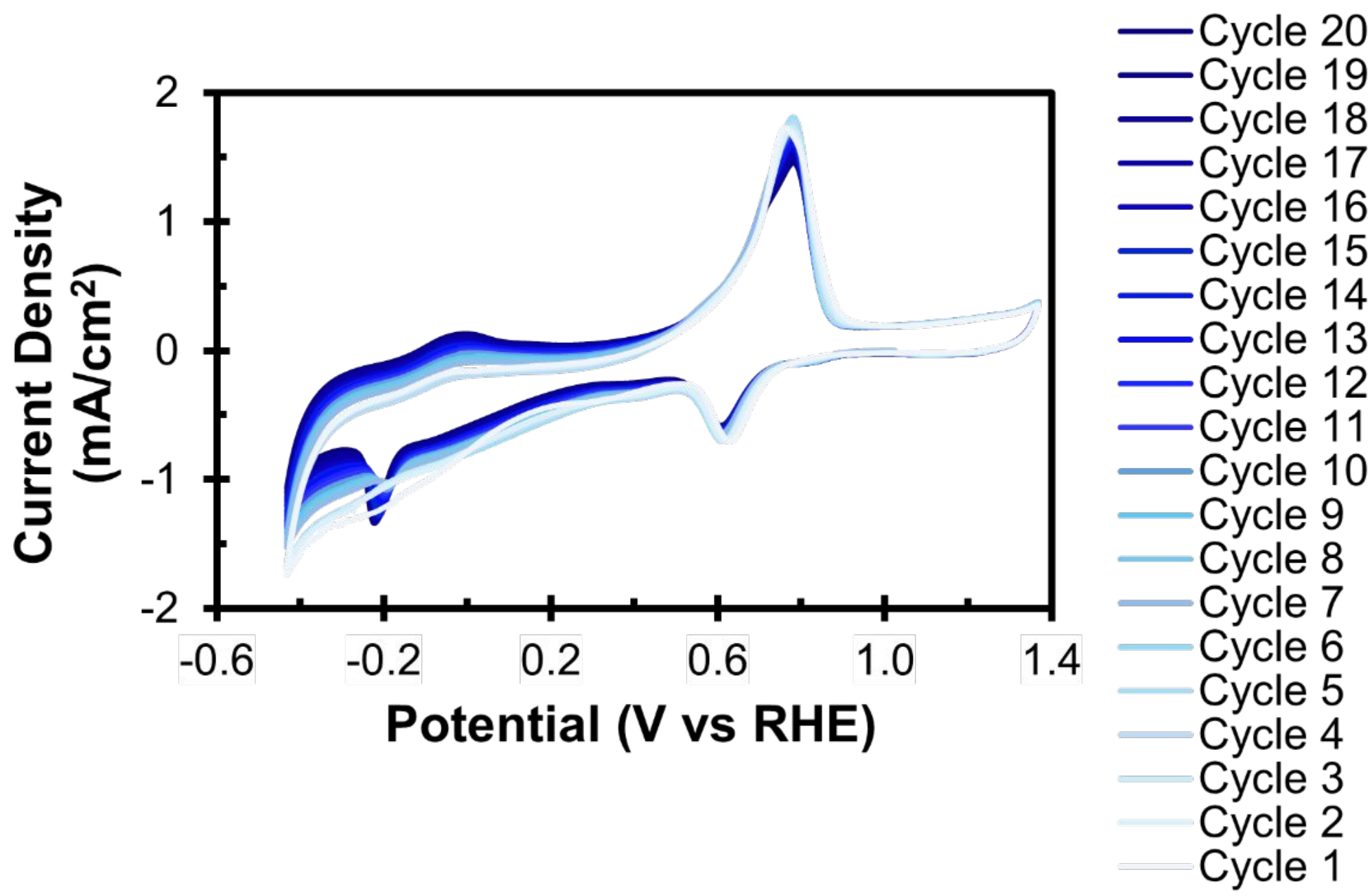

Supplementary Fig. 2 *– First 20 cycles observed during cyclic voltammetry over Pd in 0.1 M KOH after 10 mM* Ge^{4+} *is added into the electrolyte.*

Supplementary Fig. 3: Electrochemical Quartz Crystal Microbalance

The potential-dependent mass change observed during a single steady state Ge incorporation CV is shown in Supplementary Fig. 3A. Significant positive mass change is observed at potentials where Ge is underpotentially deposited onto the electrode surface, as expected. When the scan direction is reversed the mass of the electrode begins to decline. However, it does not return to the original mass. These data confirm that irreversible Ge surface incorporation occurs during CV. This mass gain was not observed in the absence of Ge^{4+}, as shown in Supplementary Fig. 3B.

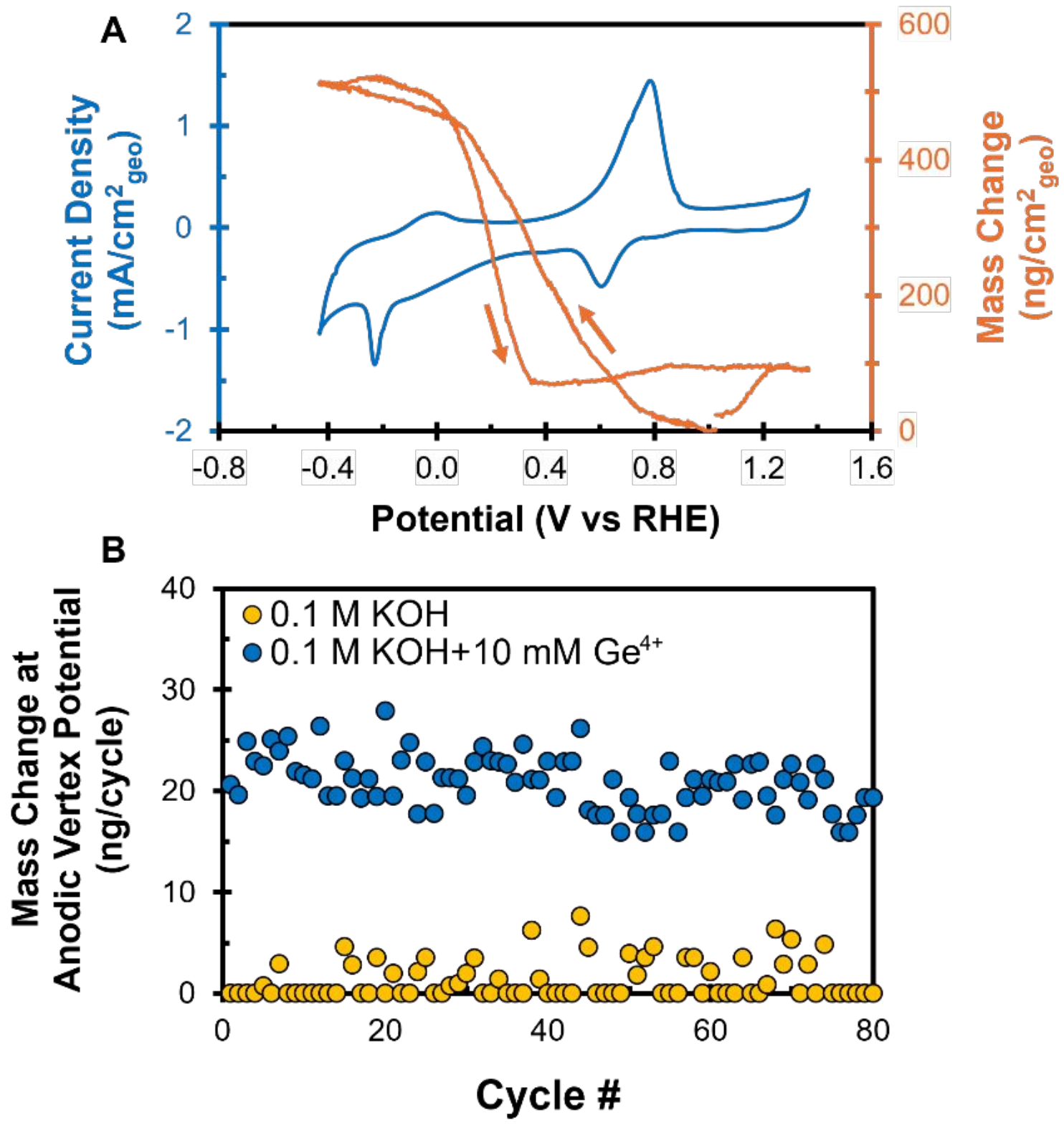

Supplementary Fig. 3 *– A) Mass observed during a steady state cyclic voltammogram over Pd in 0.1 M KOH + 10 mM* Ge^{4+}*. B) Electrochemical quartz crystal microbalance measurements during cyclic voltammetry over Pd in 0.1 M KOH with and without the addition of 10 mM* Ge^{4+}*.*

Supplementary Calculation 1: Monolayer Equivalents of Ge Deposited per Cycle

The roughness factor of the electrode is first calculated using:

$$RF = \frac{Q_{CO\ Ox,Pd} * N_A}{n * F * \theta_{CO,Pd\ in\ Pd} * N_S * 10^3} \tag{2}$$

where

RF	Roughness Factor (cm^2_{ecsa}/cm^2_{geo})	3.5
$Q_{CO\ Ox,Pd}$	CO striping charge over Pd	1.13 mC/cm^2_{geo}
N_A	Avogadro's Number	$6.022*10^{23}$ atoms/mol
n	# of electrons transferred per CO	2 mol e^-/mol CO
F	Faraday's Constant	96485 C/mol e^-
$\theta_{CO,Pd\ in\ Pd}$	Saturation Coverage of CO on Pd	0.66 molecule CO/atom Pd
N_S	Surface atom density	$1.526*10^{15}$ atoms Pd/cm^2_{ecsa}

The monolayer equivalents of Ge irreversibly incorporated per cycle is then calculated using:

$$\theta_{Ge} = \frac{\Delta m_{Ge} * N_A * 10^9}{MW_{Ge} * A_{geo} * RF * N_S} \tag{3}$$

where

θ_{Ge}	Monolayer Equivalents of Ge	
Δm_{Ge}	Average mass change per cycle	20.6 ng/cycle
MW_{Ge}	Molecular weight of Ge	72.64 g Ge/mol Ge
A_{geo}	Geometric electrode area	1 cm^2_{geo}
RF	Roughness factor	3.5

Supplementary Fig. 4: Grazing Incidence X-Ray Diffraction (GIXRD)

GIXRD was used to identify the Pd_xGe_{1-x} phase produced during electrochemical near-surface alloying. Three new Bragg reflections were observed after electrochemical cycling in the presence of Ge^{4+}. These 3 Bragg reflections are exhibited by $Pd_{25}Ge_9$, $Pd_{21}Ge_8$, and Pd_2Ge. Thus, these measurements support the notion that near-surface alloying has occurred but are unable to conclusively identify the intermetallic phase produced. However, the bulk compositions of these phases agree well with the near-surface compositions measure via XPS and the sub-surface compositions measured during the Ne-LEIS depth profile.

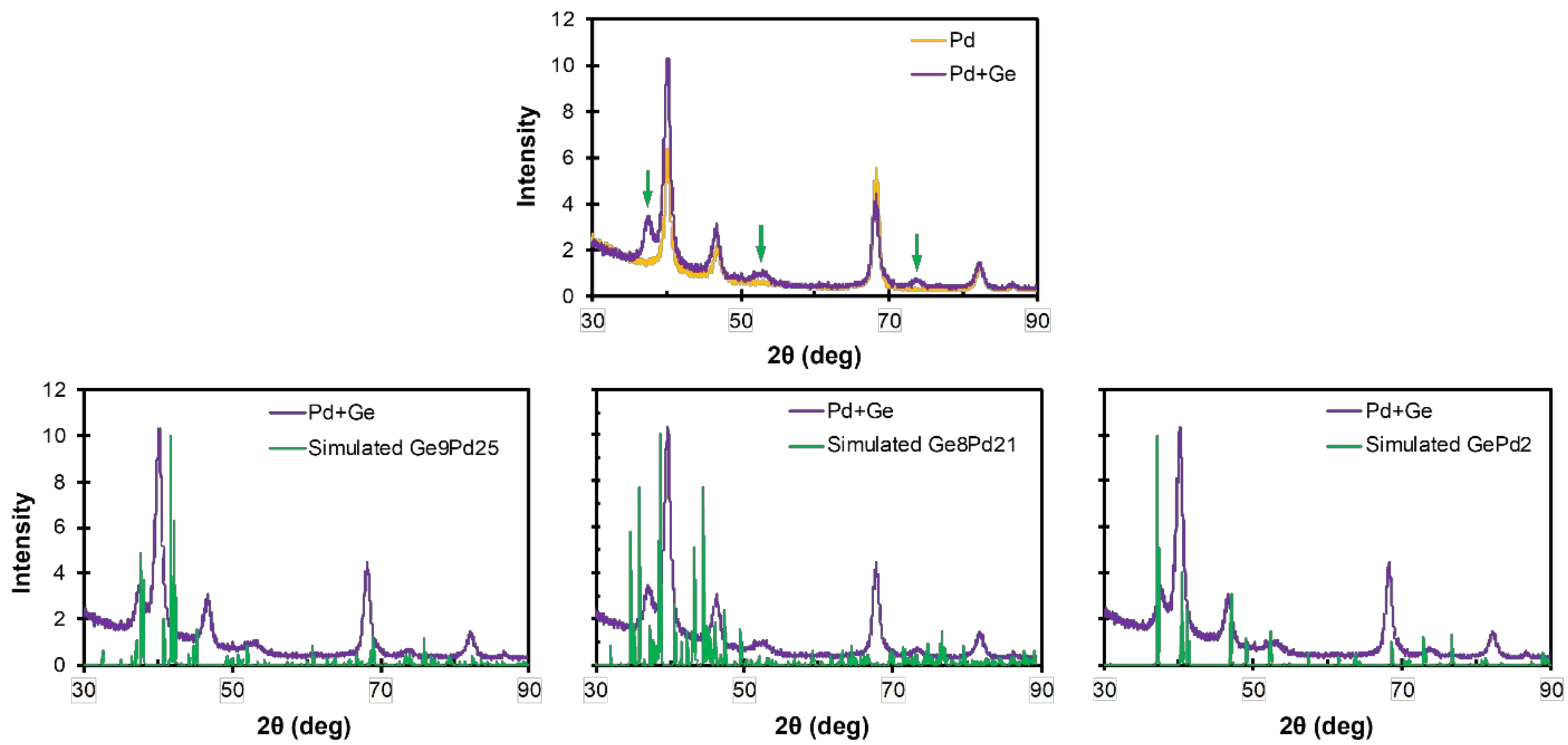

Supplementary Fig. 4 *– GIXRD of Pd before and after performing 100 Ge incorporation cycles in 0.1 M KOH + 10 mM* Ge^{4+}.

Supplementary Fig. 5 and 6: *Operando* Electrochemical EXAFS

Operando electrochemical EXAFS of the Ge K-edge was measured as a function of applied potential. Unfortunately, the signal to noise ratio was insufficient to perform accurate fitting, as shown in Supplementary Fig. 5.

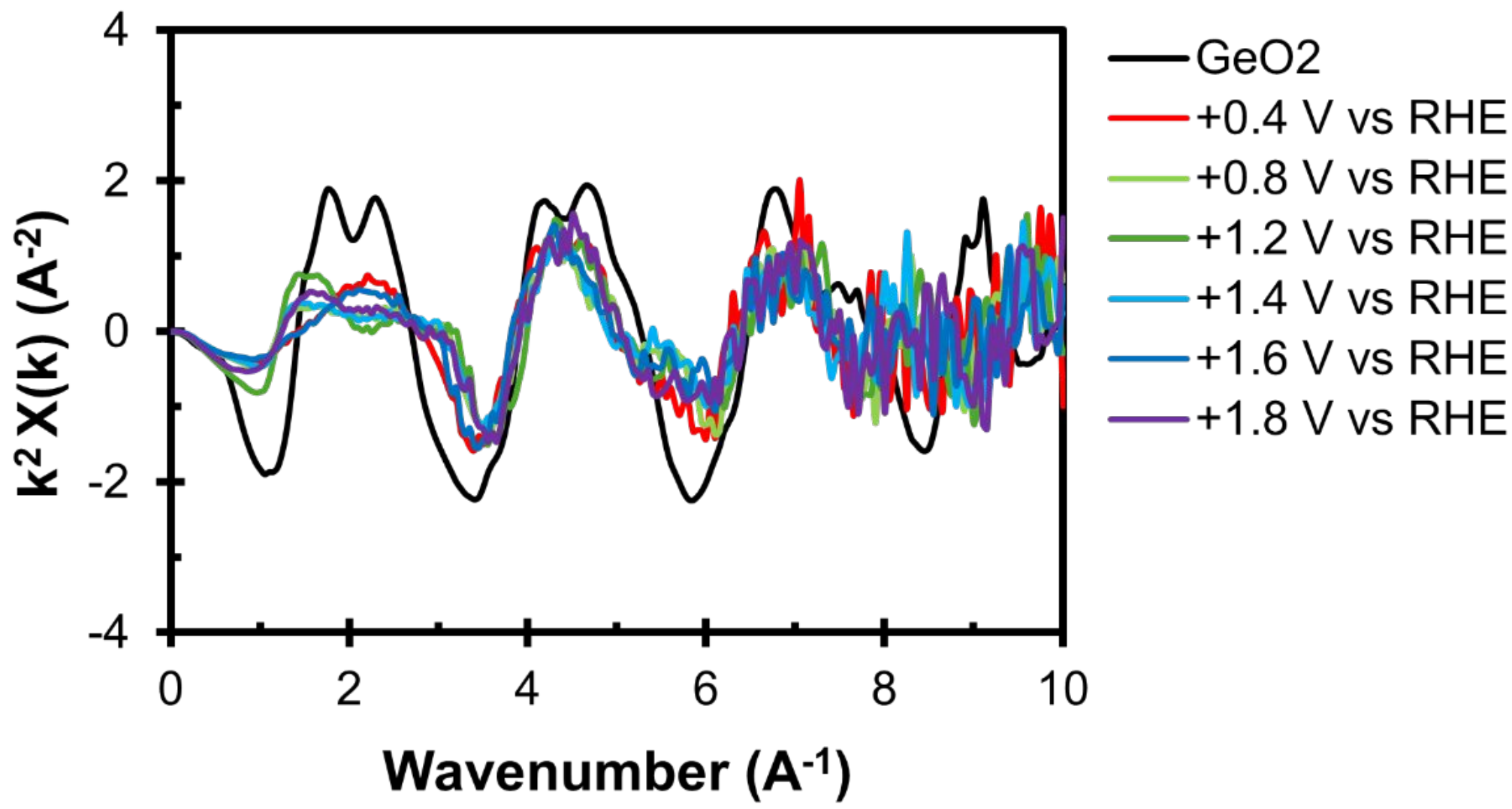

***Supplementary Fig. 5** – Operando electrochemical EXAFS of the Ge K-edge of Pd+Ge as a function of potential in 0.1 M KOH.*

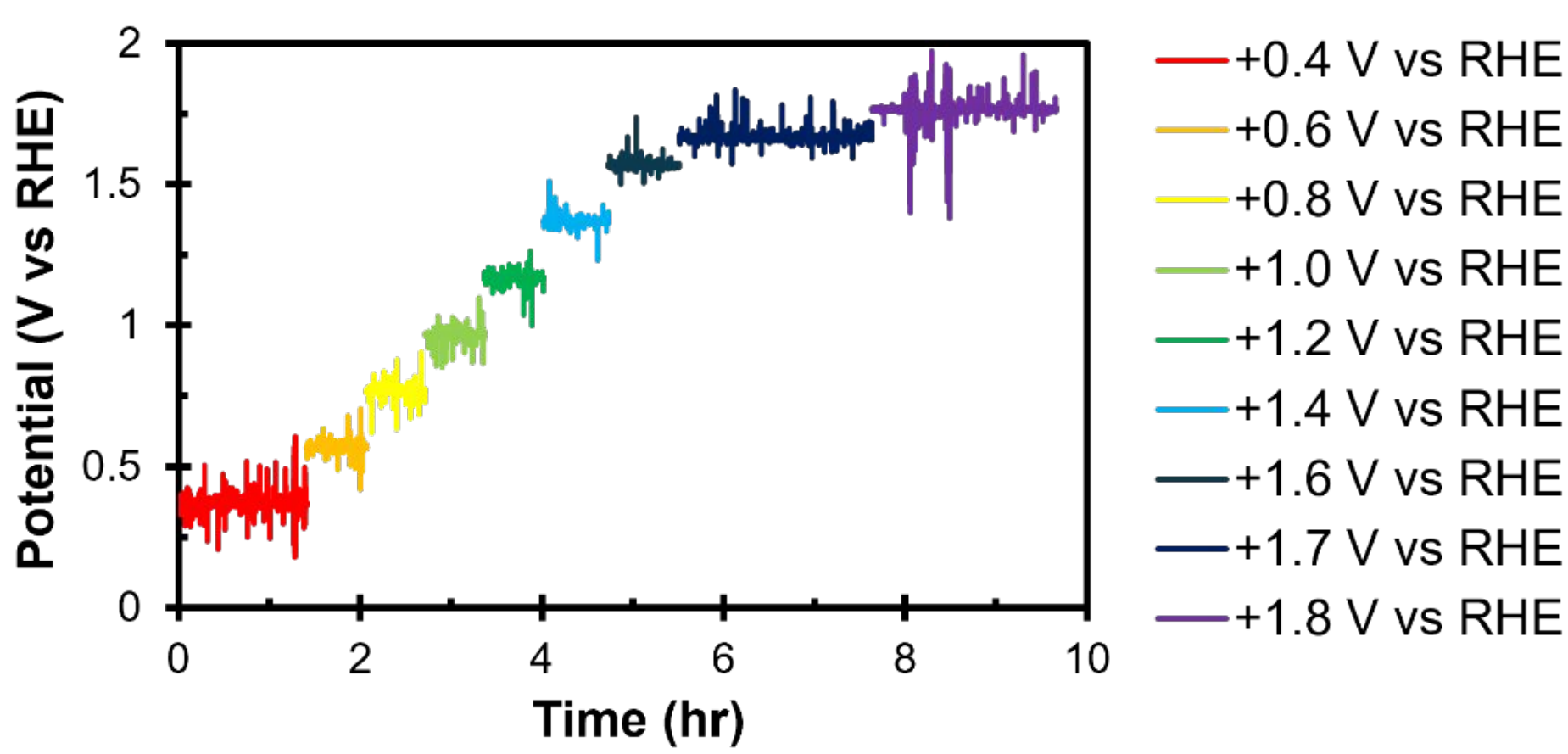

***Supplementary Fig. 6** – Chronoamperometry of Pd+Ge in 0.1 M KOH as a function of the applied potential during operando electrochemical XAS measurements.*

Supplementary Calculation 2: Surface Composition from the CO Stripping Charge

CO stripping was performed before and after electrochemical near-surface alloying. The CO stripping charges were used to calculate the surface composition of Pd+Ge using:

$$x_{Pd} = \frac{Q_{CO\ ox,Pd+Ge}\theta_{CO,Pd\ in\ Pd}}{Q_{CO\ ox,Pd}\theta_{CO,Pd\ in\ Pd+Ge}RF_{Pd+Ge}} \quad (4)$$

where

x_{Pd}	Surface Composition (at. % Pd)	
$Q_{CO\ Ox,Pd+Ge}$	CO striping charge over Pd+Ge	0.68 mC/cm^2_{geo}
$\theta_{CO,Pd\ in\ Pd}$	Saturation coverage of CO on Pd in Pd	0.66
$Q_{CO\ Ox,Pd}$	CO striping charge over Pd	1.13 mC/cm^2_{geo}
$\theta_{CO,Pd\ in\ Pd+Ge}$	Saturation coverage of CO on Pd in Pd+Ge	1.00

This is justified by the results of the calculation, which suggest that the surface composition is ~50 at. % Pd. Therefore, a CO coverage of 50% is obtained when all Pd surface sites are saturated with CO. This CO coverage is below the critical coverage required for adsorbate-adsorbate interactions to reduce the differential CO adsorption energy. Thus, complete coverage of Pd by CO in Pd+Ge can occur. Conversely, only 2/3 coverage of Pd by CO occurs for bulk Pd since adsorbate-adsorbate interactions significantly weaken the CO adsorption energy once the CO coverage exceeds 2/3.

RF_{Pd+Ge}	Roughness factor of Pd+Ge relative to Pd	0.8

Supplementary Table 1: Binding Energies of OH* and CO* Across Pd(111)+Ge and Bulk Pd_XGe_{1-X}

Supplementary Table 1 *– d-Band centers and binding energies of OH* and CO* across Pd(111)+Ge surface alloys and bulk Pd_xGe_{1-x} intermetallic alloys. The calculated d-Band centers are based on the surface Pd atom of the bare metal surface.*

**Surface**	**d-Band Center (eV)**	**OH* Binding Energy (eV)**	**CO* Binding Energy (eV)**
Pd(111)	0.00	0.81	-2.00
Pd(111)+Ge 1/2 ML Surf	-1.83	0.50	-1.03
Pd(111)+Ge 1/2 ML Sub	-1.67	0.33	-1.69
Pd(111)+Ge 1 ML Sub	-2.16	-0.62	0.11
Pd(111)+Ge 1/2 ML Surf+Sub	-2.34	0.26	-0.75
PdGe(101)	-2.84	0.00	-0.31
$Pd_{21}Ge_8$(121)	-2.05	0.45	-1.62
$Pd_{25}Ge_9$(111)	-2.36	0.20	-1.01

Supplementary Fig. 7: Binding Energy of OH* over Pd(111) and $Pd_{25}Ge_9$(111)

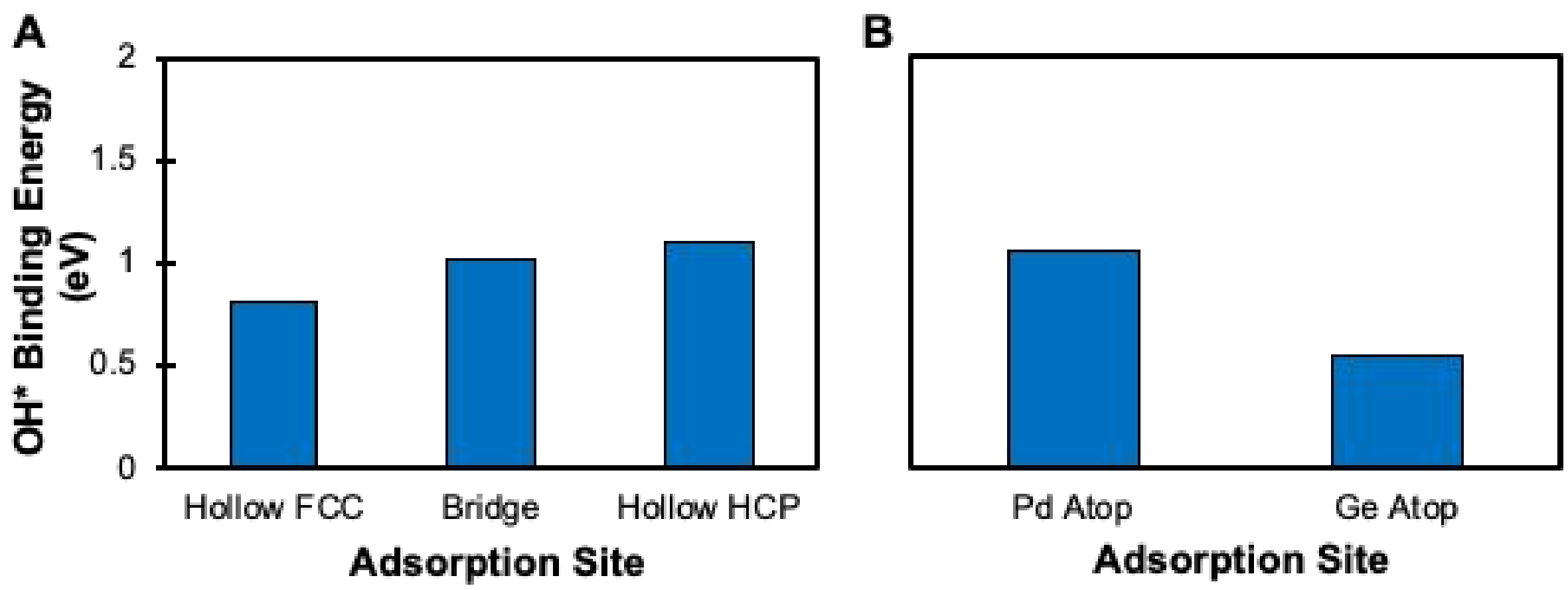

Supplementary Fig. *7 – Binding energies of OH* to various adsorption sites over: (A) Pd(111) and (B) $Pd_{25}Ge_9$(111).*

Supplementary Table 2 *– ORR limiting potentials and rate-determining steps over Pd(111) and various Pd_xGe_{1-x} surface models. Calculations performed without solvation corrections.*

Model	Ge Composition Subsurface / Surface (at. %)	Limiting Potential (V)	Rate-Determining Step
Pd(111)	0% / 0%	0.27	O* → OH*
Subsurface	50% / 0%	0.56	OH* → H_2O
Surface	0% / 50%	0.09	O* → OH*
Alloy	50% / 50%	0.23	O* → OH*
Gradient	25% / 50%	0.17	O* → OH*
